# B3Pred: A Random-Forest-Based Method for Predicting and Designing Blood–Brain Barrier Penetrating Peptides

**DOI:** 10.3390/pharmaceutics13081237

**Published:** 2021-08-11

**Authors:** Vinod Kumar, Sumeet Patiyal, Anjali Dhall, Neelam Sharma, Gajendra Pal Singh Raghava

**Affiliations:** 1 Department of Computational Biology, Indraprastha Institute of Information Technology, Okhla 110020, India; vinodporiya032@gmail.com (V.K.); sumeetp@iiitd.ac.in (S.P.); anjalid@iiitd.ac.in (A.D.); neelams@iiitd.ac.in (N.S.); 2 Bioinformatics Centre, CSIR-Institute of Microbial Technology, Sector-39A, Chandigarh 160036, India

**Keywords:** blood–brain barrier, penetrating peptides, machine learning techniques, drug delivery, prediction server

## Abstract

The blood–brain barrier is a major obstacle in treating brain-related disorders, as it does not allow the delivery of drugs into the brain. We developed a method for predicting blood–brain barrier penetrating peptides to facilitate drug delivery into the brain. These blood–brain barrier penetrating peptides (B3PPs) can act as therapeutics, as well as drug delivery agents. We trained, tested, and evaluated our models on blood–brain barrier peptides obtained from the B3Pdb database. First, we computed a wide range of peptide features. Then, we selected relevant peptide features. Finally, we developed numerous machine-learning-based models for predicting blood–brain barrier peptides using the selected features. The random-forest-based model performed the best with respect to the top 80 selected features and achieved a maximal 85.08% accuracy with an AUROC of 0.93. We also developed a webserver, B3pred, that implements our best models. It has three major modules that allow users to predict/design B3PPs and scan B3PPs in a protein sequence.

## 1. Introduction

**Figure 1:**
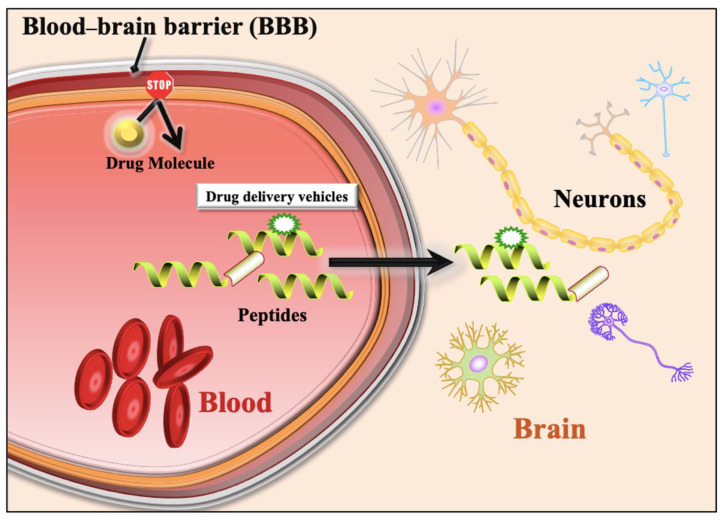
A schematic diagram shows inhibition of drug molecules entry from blood to brain due to Blood–brain barrier. It also shows entry of drug molecules from blood to brain with the support of Blood–brain barrier penetrating peptides.

The blood–brain barrier (BBB) is the primary barrier between the brain’s interstitial fluid and the blood. It is the connection between the central nervous system (CNS) and the peripheral nervous system (PNS) [1,2,3,4]. The neurovascular unit (NVU) is the structural and functional unit of the BBB, formed by neurons, macrophages, endothelial cells, astrocytes, and pericytes [5] (Figure 1). The NVU regulates the biochemical environment between the blood and the brain, which is essential for neural function. The endothelial cells of the NVU allow the entry or exit of molecules, such as glucose, amino acids, and proteins/peptides, into or from the CNS [6,7,8]. In the last few decades, researchers have made many attempts to develop drug delivery systems that can deliver drugs into the brain. Despite advances made by the scientific community in developing drug delivery systems, it is still challenging to penetrate the BBB [9].

In the past, researchers have attempted to develop peptide/protein-based drug delivery vehicles. In this approach, a major challenge is to identify peptides that can penetrate the BBB [10]. In addition, researchers are exploring peptide-based therapeutics to treat CNS-associated diseases, including neurodegenerative disorders such as Parkinson’s disease, Alzheimer’s disease [11,12], and glioblastoma [13]. This means that peptides can be used as therapeutic agents as well as drug delivery vehicles. In recent studies, numerous peptides, such as shuttle peptides [14], self-assembled peptides [15], and peptide-decorated nanoparticles [16], have been used for efficient drug delivery into the brain. Some neuropeptides are utilized as potential therapeutic targets against many neurological diseases, such as epilepsy [17,18], depression [19,20], and neuroimmune disorders [21]. Due to the low toxicity of these peptides, they may act as potential peptide-based drug candidates against neurological diseases. The major limitation of these peptide-based drugs is low bioavailability, short half-life [22], and weak penetration of the BBB [23]. For example, tumor homing peptides (THPs) [24] and cell-penetrating peptides (CPPs) [25] can be used as drug delivery vehicles [26,27]. The tumor homing peptides need a carrier to cross the BBB, while selected CPPs can directly pass through the BBB [28].

The cell-penetrating peptides are short peptides which act as molecular delivery vehicles, and are able to deliver various therapeutic molecules inside a cell [29,30]. There are CPPs that can even cross the blood–brain barrier, which are called blood–brain barrier penetrating peptides (B3PPs). These B3PPs can be used to deliver several cargo molecules (e.g., peptides/proteins, siRNA, plasmid DNA) into the brain [31,32,33,34]. Mainly, these peptides are obtained from naturally occurring proteins/peptides such as signal peptides, RNA/DNA-binding proteins, viral proteins, and antimicrobial peptides [35]. Several studies have shown that B3PPs may be synthesized chemically or designed with rDNA technology [36,37,38] to enhance the stability and half-life of the B3PPs [39]. In the past, several methods have been developed for predicting cell-penetrating peptides, such as cellPPD, SkipCPP-Pred, CPPred-RF, KELM-CPPpred, CellPPDMod, and CPPred-FL [40,41,42,43,44,45]. In addition, various methods have been developed for predicting chemical-based drug delivery vehicles to cross the blood–brain barrier [46,47,48]. In contrast, a limited attempt has been made to develop methods to predict B3PPs. Recently, Dai et al. developed an in silico method, BBPpred, to identify B3PPs [49].

In this study, we have developed a computational tool named “B3Pred” for predicting B3PPs with high reliability and precision. This method has the ability to classify BBPs vs. non-BBPs and CPPs vs. BBPs; it uses a large dataset for training and validation. We used three datasets, i.e., Dataset_1 (269 B3PPs and 269 CPPs), Dataset_2 (269 B3PPs, and 269 non-B3PPs), and Dataset_3 (269 B3PPs and 2690 non-B3PPs), for training and validation. We have used more than 9000 descriptors/features for the generation of the prediction models using several machine learning techniques, such as RF, DT, LR, XGB, SVM, and GBM.

## 2. Materials and Methods

### 2.1. Dataset Collection

In this study, we collected 465 blood–brain barrier penetrating peptides (B3PPs) from the B3Pdb database (https://webs.iiitd.edu.in/raghava/b3pdb/, accessed on 22 July 2020) [50]. We considered B3PPs having a length between 6 and 30 amino acid (AA) residues, inclusive. For the positive dataset, we collected 269 unique B3PPs. The major challenge of this type of study is to generate an authenticated negative dataset. We used three negative datasets in this study. Firstly, we collected unique 269 cell-penetrating peptides (CPPs) [51], other than B3PPs, and called them non-B3PPs or negative Dataset_1. In negative Dataset_2, we randomly generated 269 non-B3PPs from the Swiss-Prot database [52]. Our third negative dataset is ten times larger than the positive dataset, i.e., 2690 unique non-B3PPs randomly generated using the Swiss-Prot database. Finally, we combined the three datasets, i.e., Dataset_1 (269 B3PPs and 269 CPPs), Dataset_2 (269 B3PPs, and 269 non-B3PPs), and Dataset_3 (269 B3PPs and 2690 non-B3PPs).

### 2.2. Amino Acid Composition

Amino acid composition (AAC) analysis of peptides helped us to find out whether there were any amino acid compositional similarities/differences in different types of peptides. We compared the amino acid composition of B3PPs, CPPs, and randomly generated peptides. The following equation is used to calculate AAC:

(1) $$AAC_{i}=\frac{AAR_{i}}{TNR}\times 100$$

where *AAC_i_* and *AAR_i_* are the percentage composition and number of residues of type *i* in a peptide, respectively. *TNR* is the total number of residues in a peptide [53].

### 2.3. Two Sample Logo

The Two Sample Logo (TSL) tool was used to identify the amino acid preference at a specific position in the peptide sequences [54]. This tool needed an input amino acid sequence vector of fixed length, since the minimum size of peptides in all datasets was five residues; hence, we selected five residues from the N-terminal, and five amino acids from the C-terminal, of the peptide sequences. To create a fixed input vector, the N-terminus side residues and C-terminus residues were grouped together to generate a sequence of 10 amino acid residues. We used the 10-residue sequences generated from our dataset peptides to develop TSLs. To build these Two Sample Logos, we used all B3PPs and all non-B3PPs from the three different negative datasets.

### 2.4. Generation of Peptide Features

In order to calculate a wide range of features from the protein or peptide sequences, we used the Pfeature package [55]. Pfeature is used to generate thousands of features/descriptors. We computed the composition-based module of Pfeature to calculate >9000 descriptors of peptide sequences for positive and negative datasets. This module calculated fifteen types of features (AAC, DPC, RRI, DDOR, SE, SER, SEP, CTD, CeTD, PAAC, APAAC, QSO, TPC, ABC, and SOCN). The input vector of 9189 descriptors was used further for feature selection and machine learning purposes (Supplementary Table S1).

### 2.5. Feature Selection

This study used the SVC-L1 feature selection technique to extract an essential set of features from all the datasets. We chose the SVC-L1 method because it is much faster than other feature selection methods [56]. This method applies the L1 penalty to select a relevant set of features, after selecting the non-zero coefficients. SVC-L1 mainly considers regularization and the loss function. During the optimization process, the L1 regularization generates a sparse matrix by choosing some model features. The other important parameter used in this technique is the “C” parameter; its value is directly proportional to the selected features. The smaller the value of “C”, the fewer the number of features determined by the method. We chose the default value (i.e., 0.01) of the “C” parameter [57]. Using SVC-L1, 73 important features were identified from the 9189 features for Dataset_1 (B3PPs and CPPs peptides) and Dataset_2 (B3PPs and balanced non-B3PPs). Similarly, 145 features were selected for Dataset_3 (i.e., B3PPs and random non-B3PPs).

### 2.6. Feature Ranking

After selecting an important set of features, we ranked the features based on their importance in classification. The Feature-selector method is based on a decision-tree-like algorithm and uses the Light Gradient Boosting Machine (LightGBM) method [58]. It computes the rank of each feature based on the feature that is used to split the dataset across all the trees. Further, the top-most ranked features for each dataset were used in different machine learning techniques for the classification of B3PPs and non-B3PPs.

### 2.7. Machine Learning Techniques

We used several machine learning algorithms to classify B3PPs and non-B3PPs. In this study, we implemented decision tree (DT), random forest (RF), Logistic Regression (LR), k-nearest neighbors (KNN), Gaussian Naive Bayes (GNB), XGBoost (XGB), and Support Vector Classifier (SVC) machine learning classifiers. The different classification methods were implemented with the help of a python-based library known as Scikit-learn [59]. DT algorithms work based on non-parametric supervised learning models. The major aim of the classifier is to identify the output instance by learning various decision rules, provided in the form of input data [60]. The GNB method is a probabilistic classifier and builds on Bayes’ theorem. It is based on the assumption that the consecutive variable of every group follows the Gaussian (or normal) distribution [61]. Random forest is an ensemble-based classifier, which predicts a single tree as a response variable by training the number of decision trees. It also controls the overfitting of the models [62]. The LR technique is used to train the logistic/logit model, which gives the likelihood of an event happening. It applies a logistic function to predict the response variable or occurrence of a class [63]. The KNN method is an instance-based classifier. It usually collects the instances of the training dataset. Its prediction is based on the maximum number of votes given to a particular class which is closest to the nearest neighbor data point [64]. The XGB classifier uses the scalable tree boosting algorithm, in which an iterative approach is used for the prediction of the final output [65]. The SVC is developed on the library of support vector machines. It usually fits the data points provided as input features and provides the most suitable fit of a hyperplane that categorizes the data into two classes [66].

### 2.8. Cross-Validation Techniques

We used internal and external validation techniques to assess the performance of our classification models. In the past, several methods used 80:20 splitting of the complete dataset for training and validation [67,68]. In the current study, we implemented a similar strategy to evaluate our classification models. For each dataset, 80% of the data were used for training, and the remaining 20% were used for external validation. We applied 5-fold cross-validation techniques on the training dataset; this is called internal validation. In internal validation, training data are equally divided into five sets/folds in which four folds were used for training, and the fifth fold is used for testing the model (Supplementary Table S2–S4). This process is repeated five times so that each set is used once for testing. The final performance is computed by taking the average of the performance on the five sets. In the case of external validation, the performance of the best model on the training dataset was evaluated on a validation, or independent, dataset.

### 2.9. Performance Evaluation Parameters

We used standard evaluation parameters to compute the performance of the classification models. Threshold-dependent and -independent parameters were used in this study. The performance of the models was calculated using threshold-dependent parameters, such as sensitivity (Sens), accuracy (Acc), and specificity (Spec). Area Under the Receiver Operating Characteristic (AUROC) curve, a threshold-independent parameter, was used to measure the models’ performance. AUROC generates a curve by plotting sensitivity against (1-specificity) on various thresholds. Threshold-dependent parameters were computed using the given equations:

(2) $$Sensitivity=\frac{TP}{TP+FN}\times 100$$

(3) $$Specificity=\frac{TN}{TN+FP}\times 100$$

(4) $$Accuracy=\frac{TP+TN}{TP+FP+TN+FN}\times 100$$

where *TP, FP, TN*, and *FN* are true positive, false positive, true negative, and false negative predictions, respectively.

### 2.10. Webserver Implementation

We developed a webserver named “B3Pred” (https://webs.iiitd.edu.in/raghava/b3pred/, accessed on 22 February 2021) to identify blood–brain barrier penetrating peptides and non-B3PPs. We used HTML5, JAVA, CSS3, and PHP scripts to develop the front-end and back-end of the webserver. The B3Pred server is compatible with all the latest devices, such as mobiles, tablets, iMacs, and desktop computers. It mainly incorporates the predict, design, and protein scan modules.

## 3. Results

### 3.1. Amino Acid Composition Analysis

**Figure 2:**
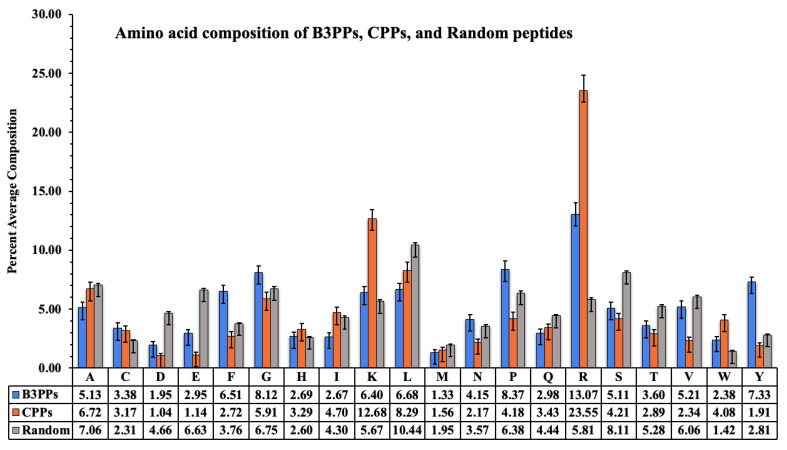
A bar graph to represent percentage amino acid composition of blood–brain barrier penetrating peptides (B3PPs), cell-penetrating peptides (CPPs), and random peptides.

The acid composition of B3PPs, CPPs, and random peptides is shown by a graph (Figure 2); the compositional difference is clearly visible. Arginine is highest in CPPs and B3PPs, which shows that it plays a crucial role in the penetration of peptides into cells. Tyrosine, an aromatic amino acid, is high in B3PPs as compared to other types of peptides. The unique amino acids proline and glycine are prevalent in B3PPs, which contrasts with other types of peptides.

### 3.2. Amino Acid Position Analysis

**Figure 3:**
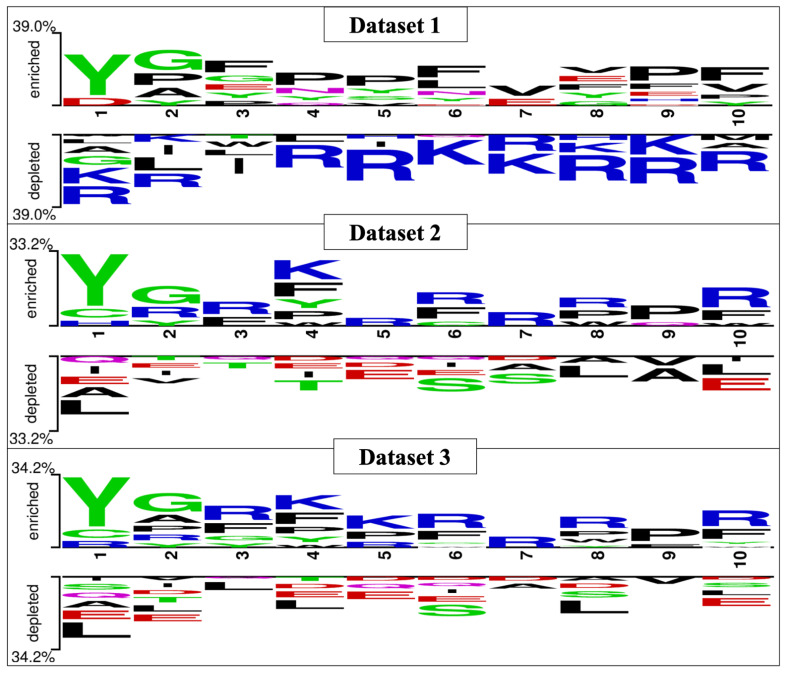
Two Sample Logo (TSL) depiction of all the three datasets (i.e., Dataset_1, Dataset_2, and Dataset_3), preferred positions for amino acids can be seen in the TSLs.

The preferential amino acid position is denoted in Figure 3, which was generated with the help of Two Sample Logo software. The preferred position of amino acids can be seen in the figure; tyrosine, glycine, and arginine are more prominent in the first three positions in B3PPs. The Two Sample Logos suggest that tyrosine, glycine, arginine, and lysine are more preferred throughout the B3PPs.

### 3.3. B3PPs Prediction Methods on Different Datasets

B3PPs prediction models were built using various machine learning techniques, such as random forest (RF), XG Boosting (XGB), Logistic Regression (LR), Support Vector Classifier (SVC), k-nearest neighbor (KNN), Gaussian Naive Bayes (GNB), and decision tree (DT) on various datasets. The best model was implemented in the webserver and standalone software. As we created three different datasets for the prediction of B3PPs, we generated 9189 peptide features by using Pfeature. These peptide features on each dataset were scrutinized and reduced by an SVC-L1 feature selection technique. The feature selection technique highlighted 73 features of Dataset_1, 73 features of Dataset_2, and 145 features of Dataset_3. After selecting features for the datasets, we developed prediction methods using different machine learning techniques. In order to classify B3PPs and CPPs, we developed models on Dataset_1, which contains 269 B3PPs and 269CPPs. Our random forest model achieved maximum performance using 73 selected features. Our RF-based method obtained an 85.12% accuracy with an AUROC of 0.92 on the training dataset, and an 84.25% accuracy with an AUROC of 0.89 on the validation dataset. KNN performed the worst and obtained a 65.58% accuracy with an AUROC of 0.74 on the training dataset, and a 50.92% accuracy with AUROC of 0.64 on the validation dataset (Table 1).

We developed classification models on Dataset_2 to classify B3PPs and non-B3PPs using different machine learning algorithms. Our RF-based model performed better than other models and achieved an 82.09% accuracy with an AUROC of 0.90 on the training dataset, and an 81.48% accuracy with an AUROC of 0.88 on the validation dataset (Table 2).

Finally, classification models were developed on Dataset_3 for discriminating B3PPs and randomly generated non-B3PPs. Our RF-based model achieved the best performance with respect to the top 80 features (Supplementary Table S5). The performance of the RF model was an 85.25% accuracy with an AUROC of 0.93 on the training dataset, and an 82.93% accuracy with an AUROC of 0.90 on the validation dataset. It was the highest-performing among all the methods on all the datasets, so we incorporated this RF model into our webserver for the prediction of the B3PPs (Table 3).

**Figure 4:**
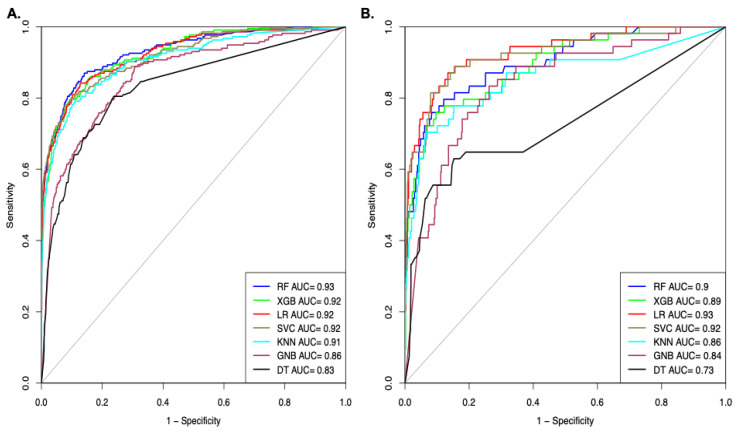
AUROC plot shows the performance of models on Dataset_3 developed using top selected features (**A**). AUROC curve for the training dataset (**B**). AUROC curve for validation dataset.

We also computed the performance of the models in terms of AUROC on Dataset_3; the models were developed using different machine learning techniques. As shown in Figure 4A, the RF-based model achieved the highest AUROC of 0.93 on the training dataset. As shown in Figure 4B, the SVC-based model achieved the maximal AUROC of 0.92 on the validation dataset (Figure 4).

### 3.4. Webserver and Standalone Software

**Figure 5:**
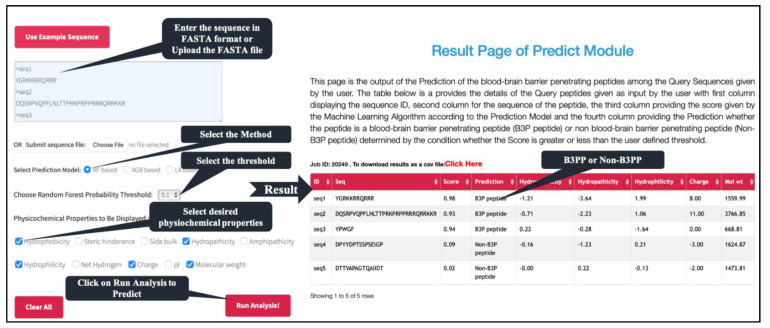
Depiction of predict module present in B3Pred webserver.

One of the major objectives of this study is to facilitate the scientific community in discovering B3PP-based drug delivery vehicles that can deliver cargo into brain tissues. Thus, we developed a standalone software as well as a web-based service to assist the researcher in finding new B3PPs or designing efficient B3PPs. Our webserver, B3Pred, has three major modules: predict, design, and scan. The predict module of B3pred allows users to predict B3PPs in a set of protein sequences submitted by the user. It allows users to select models developed on any dataset used in this study (Figure 5). The design module of B3pred was developed to discover the most promiscuous B3PPs for a given peptide. This module first generates all possible analogs of a peptide, then predicts the score for each analog. It also allows users to sort analogs, based on their score, and to select the best analog of a peptide. The scan module provides the facility to identify the B3PPs region in the user’s query protein. It allows the user to select the length of the peptide segment to be scanned in the protein sequence they submit. In addition to this web-based service, we also developed standalone software for searching B3PPs at a large scale, including searching B3PPs at the genome level.

### 3.5. Comparison with the Existing Method

It is crucial to compare this newly developed method with existing methods to understand its benefits and drawbacks. BBPpred has been developed to predict B3PPs, which is trained on 100 B3PPs and 100 non-B3PPs, and the model is tested on only 19 B3PPs and 19 non-B3PPs. On the other hand, B3Pred is trained and tested on three different datasets: Dataset_1 contains 269 B3P peptides and 269 CPPs; Dataset_2 comprises 269 B3P peptides and 269 non-B3P peptides randomly generated using the Swiss-Prot database; and Dataset_3 accommodates 269 B3P peptides and 2690 non-B3P peptides randomly generated using the Swiss-Prot database. In terms of performance, BBPpred achieved a maximal AUROC of 0.87, whereas B3Pred achieved AUROCs of 0.92, 0.90, and 0.93 on Dataset_1, Dataset_2, and Dataset_3, respectively. BBPpred only provides the prediction facility; on the other hand, B3Pred provides a prediction, design, and scan facility. In addition, B3Pred is also available as standalone software, so that users can run it on their local machine at a large scale.

## 4. Discussion and Conclusions

The blood–brain barrier (BBB) is the natural guard of the brain, which inhibits unwanted molecules from crossing into brain tissue [69]. Unfortunately, neurological disease prevalence has increased tremendously in the last few decades. Thus, there is a need to discover new drugs that can be used to treat brain-associated diseases such as Alzheimer’s disease and Parkinson’s disease. Due to advancements in technology, researchers can discover drugs to treat these disorders in vitro. One of the major hurdles in treating brain-associated disease is delivering drugs into brain tissue, as the blood–brain barrier inhibits these drug molecules from reaching this tissue [70]. The transportation or delivery of the therapeutic molecules across the barriers of the brain is the major bottleneck in treating brain tumors and CNS diseases [71].

Several in silico methods have been developed to predict and improve the delivery of therapeutic molecules that circumvent the BBB. A study has shown that D-Ala-Peptide T-amide (DAPTA), or peptide T is an antiviral peptide that can cross the blood–brain barrier. Intranasal Peptide T can be obtained from the envelope protein of the human immunodeficiency virus (HIV). This peptide shows antiviral properties, usually inhibits chemokine (CCR5) receptors, and also acts as a B3PP [72,73]. Researchers have also found that AH-D, an amphipathic α-helical BBB-penetrating peptide, can act as a therapeutic agent for deadly viruses. It is used as a direct antiviral agent (DAA) to inhibit specific viral proteins. A recent study has suggested that potential antiviral AH-D is a target against deadly viruses, such as chikungunya virus, Zika, dengue, and yellow fever, with different inhibitory and cytotoxic concentrations [74,75,76,77]. These studies show that such peptides can be helpful in viral infections, along with any neurological complications that arise due to these viruses. These peptides can be used as therapeutic substitutes for antiviral drugs which are unable to cross the brain. This may help in controlling the neurological complications that arise due to COVID-19 [78].

In the present scenario, there is the utmost need to develop an efficient prediction tool that can accurately predict the peptides that have the property of penetrating through the blood–brain barrier. To facilitate the researchers working in this area, we proposed a method named B3pred for predicting B3PPs. We have also developed a free webserver, named B3pred, and have incorporated various modules to predict, design, scan for and analyze B3PPs. We believe that our method will help in the accurate prediction of B3PPs and aid the scientific community working in this area.

## Figures and Tables

**Table 1:** The performance of classification models developed on Dataset_1 for discriminating B3PPs and CPPs; models were developed using different machine learning techniques.

Methods	Training Dataset_1					Validation Dataset_1				
	**Sens**	**Spec**	**Acc**	**AUROC**	**MCC**	**Sens**	**Spec**	**Acc**	**AUROC**	**MCC**
**RF**	86.04	84.18	82.09	0.90	0.64	75.92	87.03	81.48	0.88	0.63
**XGB**	81.39	82.32	80.93	0.88	0.62	79.63	88.89	84.25	0.88	0.68
**LR**	82.79	83.25	83.48	0.90	0.67	81.48	87.03	84.26	0.91	0.69
**SVC**	83.25	82.79	81.86	0.88	0.64	74.07	92.59	83.33	0.91	0.67
**KNN**	66.51	64.65	65.58	0.74	0.32	48.18	77.77	62.93	0.72	0.27
**GNB**	84.18	82.32	80	0.86	0.61	53.70	94.44	74.07	0.86	0.52
**DT**	78.14	75.34	73.49	0.79	0.47	74.07	70.37	72.22	0.76	0.44

**Table 2:** The performance of classification models developed on Dataset_2 for discriminating B3PPs and non-B3PPs; models were developed using different machine learning techniques.

Methods	Training Dataset_2					Validation Dataset_2				
	**Sens**	**Spec**	**Acc**	**AUROC**	**MCC**	**Sens**	**Spec**	**Acc**	**AUROC**	**MCC**
**RF**	80.57	84.18	82.09	0.90	0.64	75.92	87.03	81.48	0.88	0.63
**XGB**	80.46	81.39	80.93	0.88	0.62	79.63	88.89	84.25	0.88	0.68
**LR**	80.46	86.52	83.48	0.90	0.67	81.48	87.03	84.26	0.91	0.69
**SVC**	79.07	84.65	81.86	0.88	0.64	74.07	92.59	83.33	0.91	0.67
**KNN**	50.23	80.93	65.58	0.74	0.32	48.18	77.77	62.93	0.72	0.27
**GNB**	72.55	87.44	80	0.86	0.61	53.70	94.44	74.07	0.86	0.52
**DT**	73.02	73.95	73.49	0.79	0.47	74.07	70.37	72.22	0.76	0.44

**Table 3:** The performance of classification models developed on Dataset_3 for discriminating B3PPs and CPPs; models were developed using different machine learning techniques.

Methods	Training Dataset_3					Validation Dataset_3				
	**Sens**	**Spec**	**Acc**	**AUROC**	**MCC**	**Sens**	**Spec**	**Acc**	**AUROC**	**MCC**
**RF**	86.97	85.08	85.25	0.93	0.51	81.48	83.08	82.93	0.90	0.44
**XGB**	72.55	93.82	91.88	0.92	0.58	72.22	92.00	90.20	0.892	0.52
**LR**	80.93	89.73	88.93	0.92	0.54	83.33	89.40	88.85	0.93	0.55
**SVC**	80.00	84.75	84.32	0.90	0.45	85.18	82.15	82.43	0.90	0.45
**KNN**	83.72	80.76	81.03	0.88	0.43	79.63	78.44	78.54	0.84	0.37
**GNB**	80.46	75.74	76.20	0.84	0.35	83.33	72.67	73.65	0.86	0.34
**DT**	85.11	65.00	66.83	0.82	0.30	64.82	63.20	63.40	0.72	0.20

## Data Availability

The datasets are available at https://webs.iiitd.edu.in/raghava/b3pred/download.php (accessed on 7 July 2021).

## Supplementary Materials

The following are available online at https://www.mdpi.com/article/10.3390/pharmaceutics13081237/s1, Table S1: Description of all the 9189 features calculated using composition-based module of Pfeature; Table S2: Fold-wise performance of various machine learning algorithms on Dataset_1; Table S3: Fold-wise performance of various machine learning algorithms on Dataset_2; Table S4: Fold-wise performance of various machine learning algorithms on Dataset_3; Table S5: Top 80 features selected after implementation of SVC-L1 with their importance score calculated using feature-selector python library
